# Modeling therapy resistance via the EGFR signaling pathway

**DOI:** 10.1111/febs.14809

**Published:** 2019-03-20

**Authors:** Christina Plattner, Hubert Hackl

**Affiliations:** ^1^ Division of Bioinformatics, Biocenter Medical University of Innsbruck Austria

## Abstract

Mutations in KRAS are often associated with resistance to EGFR‐targeting antibody therapy. Using comprehensive systems analyses, GNB5 has been identified as a potential target to overcome therapy resistance targeting the EGFR signaling pathways, whereby the AKT signaling pathway (PI3K) rather than the ERK signaling pathway (RAS) might be dominantly affected. Personalized mathematical modeling and simulations of this signaling pathway/network and respective perturbations are of great utility to customize therapy for patients.

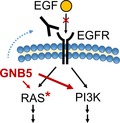

**Abbreviations**
CMS: consensus molecular subtypes
CRC: colorectal cancer
EGFR: epidermal growth factor receptor
GPCRs: G protein‐coupled receptors
GSEA: gene set enrichment analysis
mCRC: metastatic colorectal cancer
NSCLC: nonsmall cell lung cancer
ODE: ordinary differential equations
RECIST: response evaluation criteria in solid tumors
TCGA: The Cancer Genome Atlas
TKI: tyrosine kinase inhibitors

Cetuximab and panitumumab are FDA‐approved monoclonal antibodies against epidermal growth factor receptor (EGFR), which are commonly used for patients with RAS wild‐type metastatic colorectal cancer (mCRC) 1. For different indications such as nonsmall cell lung cancer (NSCLC) there are also third‐generation EGFR tyrosine kinase inhibitors targeting specific resistance mutations in the tyrosine kinase domain (T790M) approved 2. KRAS is a downstream signaling molecule of EGFR and is mutated in approximately 40% of CRC patients. However, only a small fraction of patients respond to this therapy and drug resistance remains a major issue 1. EGFR inhibitors such as cetuximab bind to the extracellular domain of EGFR, which is a transmembrane receptor tyrosine kinase. RAS‐RAF‐MAPK, PI3K‐PTEN‐AKT, and JAK/STAT are the three major downstream signaling pathways activated by EGFR. In the last decade much effort has been invested into the analyses of these signaling components and it has been shown that alterations in these downstream molecules might be involved in anti‐EGFR therapy resistance mechanisms 1, 3, 4 (Fig. 1A). So far, KRAS mutations have been the common predictors of resistance to cetuximab and panitumumab. However, recent studies, including the work from Park *et al*. 5 indicate that the KRAS status might not be sufficient to predict anti‐EGFR therapy response. Therefore, other molecules, independent of the downstream signaling components, must also be taken into account. In this issue of *The FEBS Journal,* Park *et al*. 5 were able to identify DUSP4, ETV5, GNB5, NT5E, and PHLDA1 as potential drug targets to overcome cetuximab resistance in KRAS wild‐type cells, using an comprehensive systems approach including mathematical modeling and simulation of treatment responses (Fig. 1B). Furthermore, they provide evidence that the knockdown of GNB5 increases cetuximab sensitivity even in KRAS mutant cells.

**Figure 1:**
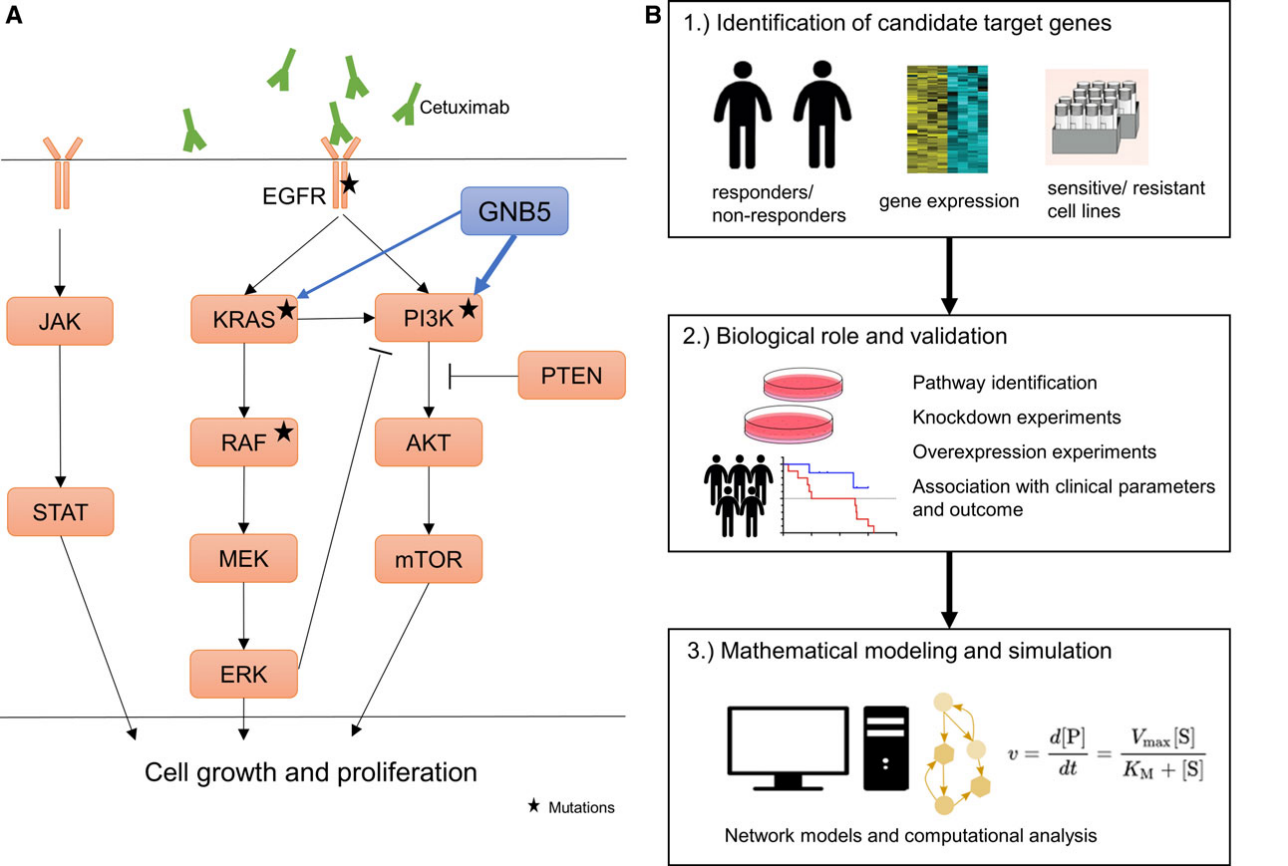
Epidermal growth factor receptor signaling pathways and common mutations associated with therapy resistance (A) and systemic approach for the identification and validation of therapy resistance targets as well as mathematical modeling and simulation of therapy response (B).

As a first step, responders and nonresponders are typically identified by progression‐free survival or according to the response evaluation criteria in solid tumors (RECIST). Significantly differentially expressed genes between these two groups may be determined using RNA sequencing or microarray analyses. Classification approaches such as random forest analyses or regularized logistic regression may be useful to narrow down the number of gene candidates with highest impact into the prediction model 6. Park *et al*. were able to verify five of the differently expressed genes, which were also significantly upregulated in cetuximab‐resistant cell lines. RNAi‐based knockdown of these candidates showed that only one candidate, GNB5, a downstream molecule of G protein‐coupled receptors (GPCRs), increased sensitivity to both the EGFR inhibitor cetuximab and the EGFR tyrosine kinase inhibitor erlotinib in KRAS mutant cell lines. Analysis in large patient cohorts, for example, data from The Cancer Genome Atlas (TCGA), is useful to associate the expression of a target with clinical parameters including CRC sidedness, TNM‐staging, microsatellite instability, consensus molecular subtypes (CMS1‐4), and with clinical outcome. Interestingly, patients with high GNB5 expression were associated with worse overall survival. Gene set enrichment analysis (GSEA) may be applied to RNA sequencing data to address if gene ontology terms/pathways, hallmarks or oncogenic signatures are enriched in tumors with high expression of the target versus tumors with low expression of the target. In the present study 5, the signaling effect was investigated in a GNB5 overexpression cell model and by using results from phosphorylation measurements, colony‐forming assays, and viability analyses. Prompted by these results, the authors suggest that GNB5 could contribute to cetuximab resistance and proliferation by dominantly affecting the Akt signaling rather than the ERK signaling.

The work by Park *et al*. 5 demonstrates that mathematical modeling [based on Michaelis–Menten kinetics and ordinary differential equations (ODE)] not only makes it possible to analyze and simulate specific signaling networks but also to investigate the effects of different perturbations 7. It is important to analyze how sensitive (or robust) the model is against variation in the estimated parameters. A model is always a simplification of reality and typically the essential factors are extracted and translated into a network, whereby the connections are derived from consensus knowledge as evident in literature and databases. In this context, the question arises whether the result (cell survival) is robust/stable against a change in the network connections, considering the variations in different cell models or cancer types. In particular, for the EGFR signaling network structural variations were considered to include or to exclude activating connection between EGFR and PI3K 8, or inhibiting connection between ERK and PI3K 9 and no changes in cell survival were observed. Sensitivity/resistance to cetuximab was shown to depend only on KRAS mutation, but could be reversed by GNB5 overexpression or GNB5 knockdown, respectively 5. Logical (Boolean) modeling 8, 10 could be an alternative in testing changes of individual factors or rules within a signaling network topology, especially patient/cell type‐specific adaptations are of interest.

However, a number of open questions still remain to be clarified, for example, if targets can be identified, which are contributing to the resistance mechanism of other tyrosine kinase inhibitors (TKI). This may include also factors from the tumor microenvironment able to rescue cancer cells from kinase inhibitors, as shown by (phospho)proteome profiling 11. Especially in the investigation of signaling networks, this technology in combination with phenotypic responses based on perturbation experiments provides a useful extension for network inference and modeling 7. Since recent observations show that targeting the EGFR signaling pathway can also trigger immunogenic cell death 12 or influence the tumor immune environment via the JAK/STAT3 axis, possible effects for immunotherapy or combination therapy are also of great interest.

Ultimately, systemic analysis and, in particular, personalized mathematical models—for example, taking into account the KRAS mutation status or the activation of EGFR variants of a patient—and corresponding simulations (validated, at least in some cases, by the treatment of patient‐related tumor organoid models), could help to customize therapy for each patient, and that is exactly what we expect from precision medicine.

## Conflict of interest

The authors declare no conflict of interest.
